# Change in the P300 index – a pilot randomized controlled trial of low-frequency electrical stimulation of acupuncture points in middle-aged men and women

**DOI:** 10.1186/s12906-017-1754-8

**Published:** 2017-05-03

**Authors:** Kwang-Ho Choi, O Sang Kwon, Seong Jin Cho, Sanghun Lee, Seok-Yun Kang, Yeon Hee Ryu

**Affiliations:** KM Fundamental Research Division, Korea Institute of Oriental Medicine, 1672 Yuseong-daero, Yuseong-Gu, Daejeon, 305-811 South Korea

**Keywords:** Low-frequency electrical stimulation, P300, BL62, KI6, Sex difference, Double-blind

## Abstract

**Background:**

The P300 is a major index used to evaluate improvements in brain function. Although a few studies have reported evaluating the effectiveness of manual acupuncture or electro-acupuncture by monitoring the P300, research in this field is not yet very active. The aim of this study was to investigate the effects of periodic low-frequency electrical stimulation applied to BL62 and KI6 on brain activity by analyzing the P300.

**Method:**

The study was conducted as a randomized double-blind test of 55 subjects in their 50s, including 26 males and 29 females. Each subject received 12 sessions of stimulation over a one-month period. In each session, low-frequency electrical stimulation at an average of 24 μA and 2 Hz was applied to the acupuncture points BL62 and KI6, and event-related potentials (ERPs) were measured before the first session and after the last session of the electrical stimulation.

**Results:**

The results of a chi-square test indicated that the double-blind test was conducted correctly. Compared to the Sham group, all the subjects in the Real stimulation group showed a tendency toward a decreasing P300 latency and increasing P300 amplitude after all 12 sessions of stimulation. In the women, the amplitude significantly increased at Fz, Fcz, Cz, Cpz, and Pz.

**Conclusions:**

With this experiment, the low-frequency electrical stimulation of two acupuncture points (BL62 and K16) was confirmed to have a positive influence on the prevention of natural cerebral aging.

**Trial registration:**

This study was registered at the Clinical Research Information Service (CRIS) of the National Research Institute of Health (https://cris.nih.go.kr/cris/search/search_result_st01_en.jsp?, Registration Number: KCT0001940). The date of registration was June 9, 2016.

## Background

Event-related potentials (ERPs) are evoked potentials observed during electroencephalogram (EEG) measurements immediately after discontinuous sensory stimulation. From ERPs, we can obtain information related to brain activity, such as sensory, cognitive, and motor events. The brain activity information that can be obtained from ERPs includes sensory-evoked components (the P50 and N100) and cognitive-related components (the P300 and N400), one of which, the P300, is a positive potential that appears approximately 300 ms after auditory or visual stimulation [1]. This component was first observed in 1965 by Sutton et al. and was generated when a subject did not recognize the type of stimulation [2]. At present, the peak generated when a subject recognizes an auditory or visual stimulus is called the P3b, and the peak generated by an unexpected distracter is called the P3a. The distinction between the P3a and P3b remains controversial, and these components have been known to differ depending on task difficulty [3].

The P300 is an important index that is used in clinical fields to evaluate reductions in memory and other cognitive functions and has been known to show decreased amplitude and delayed latency with aging. A decreased amplitude and delayed latency of the P300 indicate a general slowing of cognitive processes due to neurodegeneration or other disease-related causes [4]. To study the mechanism that generates the P300, studies that track the signal source have been conducted, including studies in which information measured on the scalp is used to determine the site of signal generation [5] and those in which generation sites are directly identified by implanting electrodes within the brains of patients with epilepsy [6]. Lesion studies have shown that P300 generation is affected by damage to the temporo-parietal junction, and the inferior parietal lobe, supramarginal gyrus and hippocampus are already known to be very important in P300 induction. However, it is assumed that P300 induction is affected by complex influences in the brain; therefore, the identification of the source of the P300 is still controversial [7].

With the development of medical technology, society is aging. Aging is related to an increase in senile brain disorders, and this increase currently poses an unavoidable problem. According to recent reports, the number of patients with Alzheimer’s dementia is expected to reach 13,500,000 by 2050 in the United States alone and will become a major problem around the world over time [8]. Efforts to improve the treatment and prevention of this affliction have been made for many years, and alternative medicine has recently been applied as a measure to supplement traditional treatments. Alternative medicine is widely used with patients suffering from diseases that are considered hopeless by current medical conventions or from chronic diseases that are seldom treated with conventional approaches [9, 10].

Electro-acupuncture (EA), used in alternative medicine, can be a good method to reduce the problem of senile brain disorders in an aging society because EA treatment can be implemented without surgery and is easy to access [11, 12]. Preliminary studies have shown that among the acupuncture points, BL62 and KI6 are effective for insomnia [13] and dementia [14]. Because insomnia is associated with brain disorders such as dementia [15] and cognitive impairment [16], BL62 and KI6 stimuli are likely to treat brain disorders in alternative medicine.

Therefore, this study evaluated the P300, a major index used to assess cognitive function, to investigate the effect of regular, non-invasive electrical stimulation of acupuncture points of the foot on cognitive function. Additionally, based on previous studies indicating that cognitive function may be different according to gender, sex differences in the observed P300 changes were assessed to confirm the results regarding sex reported in previous ERP experiments [17, 18].

## Methods

### Subjects

Candidate subjects, who were healthy men and women in their 50s, were recruited through advertisements in the district of Jeonmin-dong, Daejeon-si, South Korea between January and June, 2012. Candidates were screened to ascertain their health status.

**Fig. 1:**
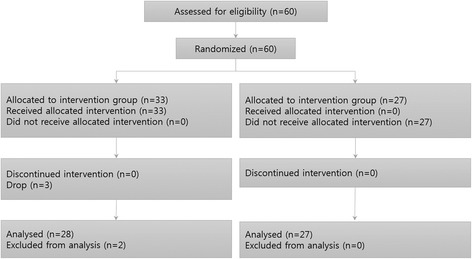
Participant flowchart. A total of 60 subjects were recruited. Of the intervention group, 30 were excluded, among whom three subjects who were dropped due to the discontinuation of the participant. Two subjects were excluded from the analysis because the data from these subjects were considered problematic due to the subject’s sleepiness or the malfunction of the machine

Candidates who met all of the following inclusion criteria were enrolled: 1) healthy males and females in their 50s or 60s; 2) individuals who agreed to voluntarily participate in the clinical trial and sign the consent form. Candidates who met any one of the following exclusion criteria were excluded: suffering from 1) an autoimmune disease; 2) an allergic skin disease; 3) a brain disorder; or 4) symptoms that could affect an electromyogram of the head or 5) the neck and face; 6) had major surgery or a critical illness within the past year; 7) had a metal prosthesis implanted in the body; 8) taking or planning to take medication; 9) unable to maintain a sitting position for one hour and thus unsuitable for electrical stimulation therapy; 10) unable to complete a legal document; or 11) unsuitable to participate in the study based on the investigator’s judgment. A total of 57 subjects (27 male; 30 female) participated in the study, and the final results were analyzed with the data from 55 subjects (26 male; 29 female) after 2 subjects were excluded (1 male; 1 female). The data of these subjects were considered problematic due to the subject’s sleepiness or to the malfunction of the machine (Fig. 1, Table 1).

**Table 1:** Demographic characteristics of the participants and the average intensity of the electrical stimulation applied

Stimulus	Real		Sham	
Sex	Male	Female	Male	Female
Number of participants	14	14	12	15
Age (Years)	55.14 ± 2.74	53.77 ± 2.71	56.00 ± 2.09	53.73 ± 3.51
Height (Cm)	170.93 ± 5.34	158.64 ± 4.11	170.50 ± 5.02	158.40 ± 4.19
Weight (Kg)	71.79 ± 8.05	57.79 ± 6.99	70.08 ± 9.94	56.93 ± 6.50
Intensity of stimulus (**µ**A)	27.00 ± 13.43	26.15 ± 11.74	21.18 ± 12.29	25.39 ± 11.43

### Low-frequency electrical stimulation

**Fig. 2:**
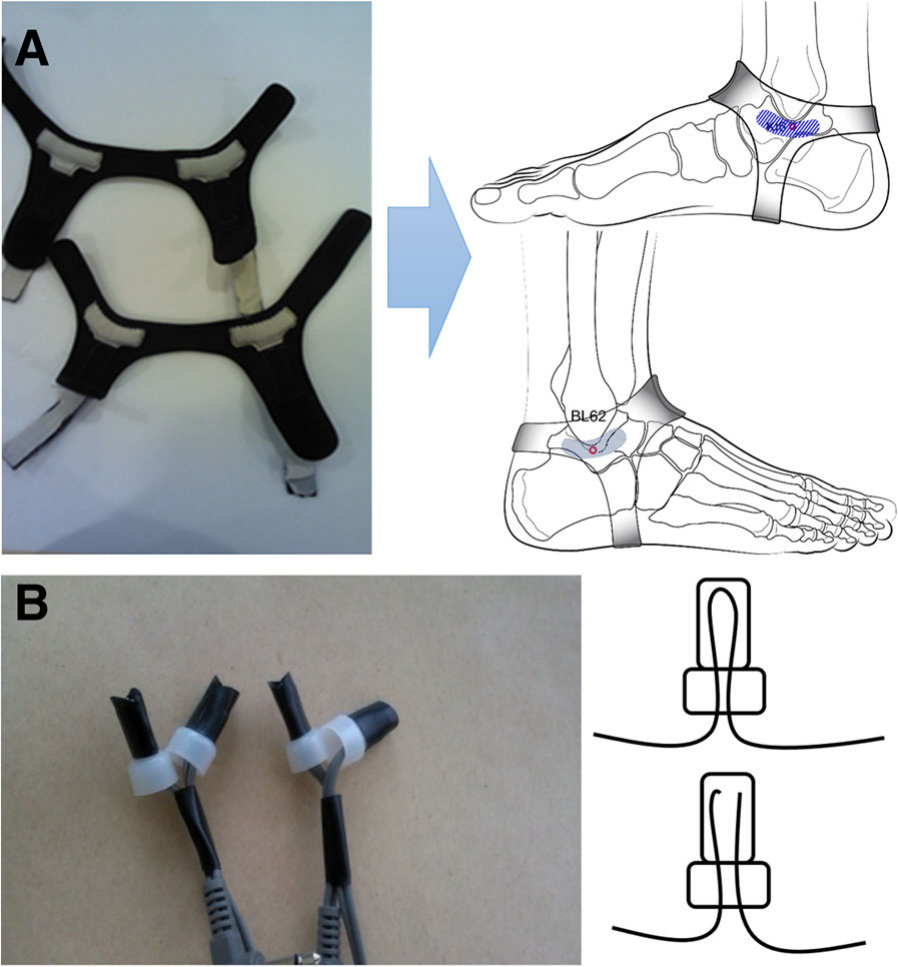
The bands (**a**) and wires (**b**) used in this study. **a** Applying low-frequency electrical stimulation at the acupuncture points BL62 and KI6 to a participant while measuring electrical current. The low-frequency electrical stimulation was applied through a band specifically designed for selective stimulation at BL62 and KI6 in conjunction with the simultaneous measurement of electrical current. **b** Electrical wires used for low-frequency electrical stimulation. Wires with or without a cut were made in an indistinguishable fashion to maintain the double-blind condition

A doctor who was licensed and certified in Korean Oriental Medicine applied non-invasive low-frequency electrical stimulation to the bilateral target acupuncture points BL62 and KI6 through a custom-built band (low-frequency electrical stimulation band, TrekSta, Korea) made of stainless steel, which was worn on the subject’s ankle (Fig. 2). The stimulation was generated by a Pulse Generator (PG-306, SUZUKI IRYOKI, Japan) and was applied to each subject for 30 min at a frequency of 2 Hz based on the results of a prior study [14]. The electrical stimulation time was set at 15 min to 30 min for sufficient stimulation. The total amount of electrical stimulation was measured using a Precision Multi-meter (8846A, FLUKE, USA) and recorded (Table 1).

### Randomized double-blind test

A total of 60 wires were prepared for the low-frequency electrical stimulation. Half of the wires were assigned to the male group, and the other half were assigned to the female group. Among the 30 wires for each group, 15 were cut prior to applying the same cover that was applied to the other 15 working wires; thus, the subjects could not see which type they were randomly assigned (Fig. 2). Furthermore, to prevent the subjects from feeling the electrical stimulation, the maximum current that could not be perceived by each subject was recorded before the study was initiated, and this maximum imperceptible current was applied to each subject on the predefined acupuncture points three times a week (for a total of 12 times over 4 weeks). Each stimulation lasted for 30 min and used the same current level. Therefore, the study was conducted under double-blind conditions, wherein neither the investigator nor the subject knew whether they gave or received electrical stimulation. The investigator also kept a record of whether the subject felt the electrical stimulation after each treatment.

### Electroencephalogram measurement

The EEG was measured using a 32-channel encephalograph, WEEG-32 (LXE3232-RF, LAXTHA, Korea). The results were collected at a sampling rate of 256 Hz, filtered from 0.7 ~ 47 Hz, and saved on a computer after 16-bit analog-to-digital (AD) conversion. Thirty-two electrodes were attached on the following locations: Fpz, Fp1, Fp2, F7, Ft7, T7, Tp7, P7, F3, Fc3, C3, Cp3, P3, F8, Ft8, T8, Tp8, P8, F4, Fc4, C4, Cp4, P4, Afz, Fz, Fcz, Cz, Cpz, Pz, O1, Oz, and O2 according to the international Modified Combinatorial Nomenclature (MCN) system. Control electrodes were placed behind the right ear, and the ground was placed at the back of the neck. The electrodes used in this study were dish-shaped disks covered with gold, and they were attached to the scalp using EEG glue. To make sure they were firmly secured to the surface of the scalp and to prevent the glue from drying quickly, gauze was placed on top of the electrodes.

Background EEG and ERPs were measured before the first treatment and after the last treatment of low-frequency electrical stimulation to the target acupuncture points. Prior to the EEG measurement, the subjects were given 30 min of resting time in a quiet environment where they sat on a chair any physical movement was restricted. First, background EEG was measured for 5 min with the subject’s eyes closed. Then, after 10 min of resting time, ERPs were measured for 5 min. During the measurement of the ERPs, standard stimulation and target stimulation were applied at a 3:1 ratio in random order according to the oddball paradigm. The standard stimulation and target stimulation used 1000 Hz and 2000 Hz, respectively. A total of 600 auditory tones (57 dB SPL) were presented to the subjects through a speaker. To help the subjects perceive the target stimuli, the subjects were exposed to each sound prior to the ERP measurement and asked to count the target stimuli in their minds.

### Sample size determination

This study was an investigation of the mechanism in the brain of low-frequency electrical stimulation on the ankle; therefore, this study did not follow the general method for calculating sample size. The current study was designed as a pilot to provide the initial data required to perform the power calculation necessary for a large-scale, randomized controlled trial. The sample size of 30 subjects in each group was chosen as the minimum sample size for statistical significance in a univariate analysis considering the subject attrition rate.

### Outcomes

The recorded data were analyzed using an analysis program (Telescan, LAXTHA, Korea). From the recorded data of each subject, 30 sets of 2-s-long signals were randomly extracted from the signals without any noise to use for the background potential analysis. The mean power spectrum amplitude and the mean dominant frequency of the slow alpha waves (8–10 Hz) were computed. Among the ERP signals measured from 0.1 s before to 0.6 s after the stimulation, only the data with an amplitude difference of less than 100 were selected to calculate the mean values, and then the amplitude and latency of the P300 were analyzed. The P300 amplitude was defined as the difference between the highest peak value of the P300 and the mean value at 0.1 s before the target stimulation. The P300 latency was defined as the position at which the P300 value was obtained after the target stimulus was applied. Only the channels Fz, Fcz, Cz, Cpz and Pz were selected for the data analysis. The primary outcomes were the P300 amplitude and latency, and the secondary outcomes were the slow alpha mean frequency and power.

### Statistical analysis

For statistical analysis, ANCOVA was used to compare the groups using the SAS program (SAS 9.1.3, SAS Inc., USA).

## Results

### P300 latency and amplitude

When the changes in the P300 were measured by comparing ERPs before and after the low-frequency electrical stimulation, an increase in amplitude was observed at all of the analyzed channels in all subjects: Fz (F = 2.06, *p* = 0.158), Fcz (F = 1.93, *p* = 0.170) Cz (F = 1.91, *p* = 0.174), Cpz (F = 4.41, *p* = 0.041), and Pz (F = 3.79, *p* = 0.057). In particular, the change at the Cpz channel in the test group (Real) was found to be significant compared to that in the control group (Sham) (*p* = 0.041). Although the latency of the P300 tended to be shifted forward at channels Fz, Fcz, Cz, and Pz in the Real group, no significant differences were observed compared to the Sham group (Table 2).

**Table 2:** Mean and standard error of the P300 latency and amplitude obtained from all subjects

		Total (*n* = 55)						Group difference (AS-NS)	*p*-value
		Sham (*n* = 27)			Real (*n* = 28)				
		Before	After	Difference	Before	After	Difference		
Latency of P300 (sec)	Fz	0.31 ± 0.06	0.31 ± 0.07	0.00	0.32 ± 0.07	0.30 ± 0.07	−0.02	−0.02	0.39
	Fcz	0.30 ± 0.07	0.29 ± 0.06	−0.01	0.32 ± 0.07	0.30 ± .007	−0.02	−0.01	0.69
	Cz	0.30 ± 0.06	0.28 ± 0.05	−0.02	0.32 ± 0.07	0.30 ± 0.07	−0.02	0.00	0.35
	Cpz	0.30 ± 0.06	0.27 ± 0.07	−0.03	0.31 ± 0.08	0.31 ± 0.07	0.00	0.03	0.13
	Pz	0.31 ± 0.07	0.28 ± 0.08	−0.03	0.31 ± 0.09	0.30 ± 0.08	−0.01	0.02	0.05
Amplitude of P300 (μV)	Fz	3.49 ± 1.35	3.37 ± 1.91	−0.12	3.76 ± 2.24	4.28 ± 2.53	0.52	0.64	0.16
	Fcz	3.28 ± 1.40	3.46 ± 1.90	0.18	3.59 ± 2.05	4.41 ± 2.56	0.82	0.64	0.17
	Cz	3.09 ± 1.23	3.35 ± 1.98	0.26	3.28 ± 1.87	4.17 ± 2.27	0.89	0.63	0.17
	Cpz	2.92 ± 1.23	2.78 ± 1.79	−0.14	3.11 ± 1.93	3.80 ± 2.03	0.69	0.83	*0.04*
	Pz	2.70 ± 1.24	2.51 ± 1.53	−0.19	2.81 ± 1.70	3.32 ± 1.81	0.51	0.70	0.06

The differences between the groups were calculated by subtracting the change from before and after the stimulation of the Sham group from that of the Real group, which shows the extent of the changes in the Real group compared with those in the Sham group. Compared to the Sham group, the Real group showed a tendency toward an increase in the P300 amplitude, particularly in channel Cpz (F = 4.41, *p* = 0.04)

Italicized data: A significant increase in P300 amplitude

The male group showed a tendency toward a reduced P300 latency at the Fz, Fcz, Cz, and Pz channels when comparing before and after stimulation. Although larger P300 amplitudes were detected at these channels, no significant differences were observed compared to the Sham group. In the female group, a shorter latency was observed at Fz, Fcz, and Cz, but no significant differences were detected compared to the Sham group. In contrast, in the Real group, the amplitude showed a significant increase at all the analyzed channels: Fz (F = 4.26, *p* = 0.049), Fcz (F = 5.23, *p* = 0.031), Cz (F = 4.63, *p* = 0.041), Cpz (F = 4.94, *p* = 0.035), and Pz (F = 6.49, *p* = 0.017)–compared to the corresponding values of the Sham group (Table 3).

**Table 3:** Mean and standard error of the P300 latency and amplitude in the female group

		Female(*n* = 29)						Group difference (AS-NS)	*p*-value
		Sham(*n* = 15)			Real(*n* = 14)				
		Before	After	Difference	Before	After	Difference		
Latency of P300 (sec)	Fz	0.31 ± 0.06	0.32 ± 0.07	0.01	0.32 ± 0.07	0.31 ± 0.07	−0.01	−0.02	0.39
	Fcz	0.29 ± 0.07	0.29 ± 0.06	0.00	0.32 ± 0.07	0.31 ± 0.07	−0.01	−0.01	0.69
	Cz	0.29 ± 0.06	0.28 ± 0.05	−0.01	0.32 ± 0.07	0.32 ± 0.06	0.00	0.01	0.35
	Cpz	0.29 ± 0.06	0.28 ± 0.07	−0.01	0.31 ± 0.09	0.32 ± 0.07	0.01	0.02	0.13
	Pz	0.32 ± 0.07	0.28 ± 0.07	−0.04	0.31 ± 0.08	0.33 ± 0.07	0.02	0.06	0.05
Amplitude of P300 (μV)	Fz	3.75 ± 1.66	3.34 ± 1.66	−0.41	4.06 ± 2.57	4.83 ± 3.15	0.77	1.18	*0.05*
	Fcz	3.48 ± 1.80	3.17 ± 1.49	−0.31	3.93 ± 2.35	5.09 ± 3.24	1.16	1.47	*0.03*
	Cz	3.30 ± 1.58	3.14 ± 1.56	−0.16	3.64 ± 2.15	4.74 ± 2.86	1.10	1.26	*0.04*
	Cpz	3.14 ± 1.56	2.91 ± 1.62	−0.23	3.49 ± 2.25	4.42 ± 2.51	0.93	1.16	*0.04*
	Pz	2.96 ± 1.53	2.59 ± 1.48	−0.37	3.08 ± 2.02	3.95 ± 2.18	0.87	1.24	*0.02*

In the female group, the Real group exhibited a significant increase at all five analyzed channels around the parietal lobe: Fz (F = 4.26, *p* = 0.049), Fcz (F = 5.23 *p* = 0.031), Cz (F = 4.63, *p* = 0.041), Cpz (F = 4.94 *p* = 0.035), and Pz (F = 6.49 *p* = 0.017) compared with the corresponding values of the Sham group

Italicized data: A significant increase in P300 amplitude

**Fig. 3:**
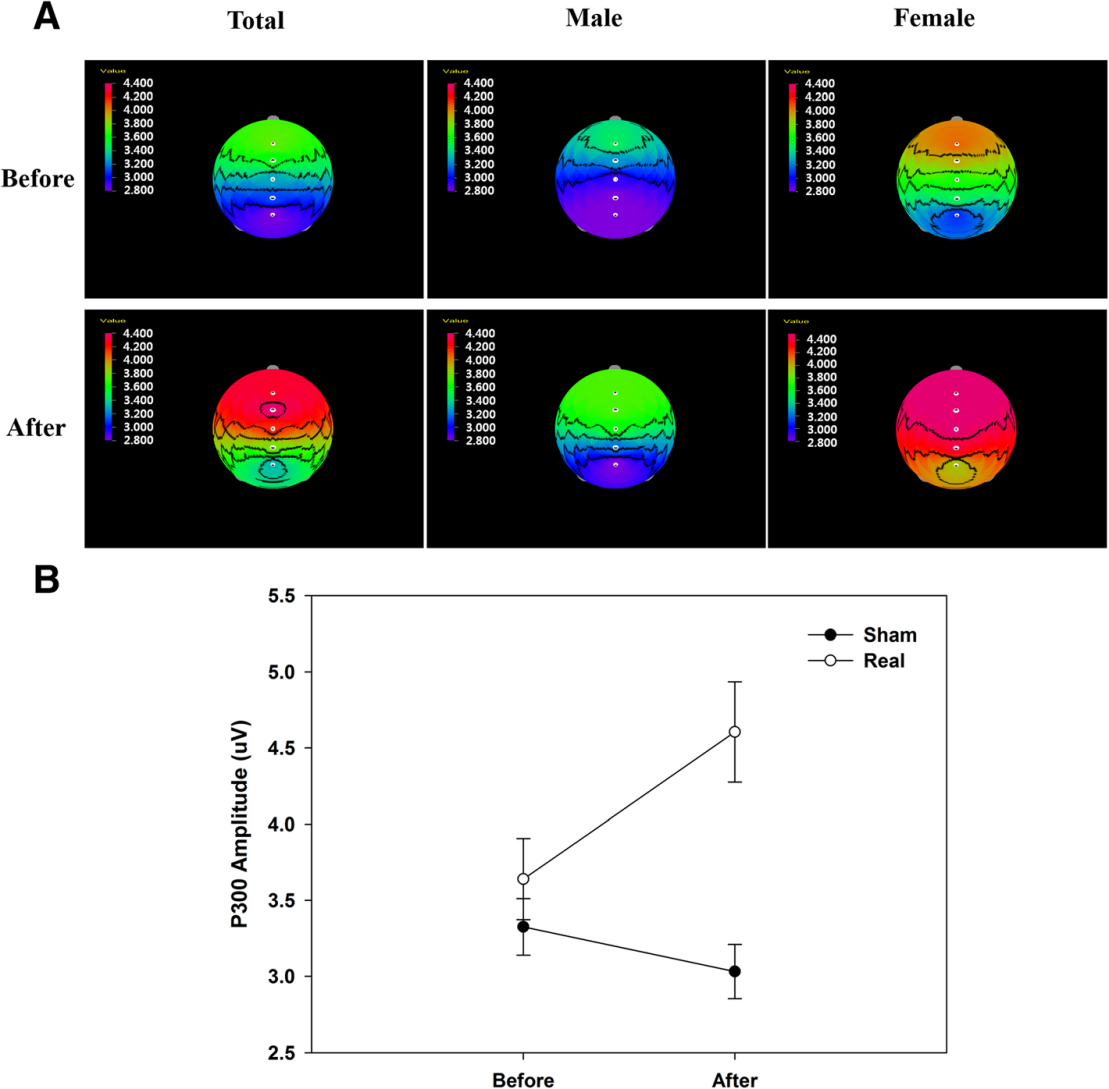
P300 amplitude change after electrical stimulation. **a** ERP topographical maps showing (the differences in) the P300 amplitude obtained from the male and female subjects before and after electrical stimulation. Power was found to increase around the parietal lobe after stimulation in both the female and male groups. In particular, changes around the frontal lobe and parietal lobe were more significant in the female group than in the male group. **b** Total P300 amplitude difference in the females. The results from five channels were averaged, demonstrating the differences in the P300 amplitude before and after the low-frequency electrical stimulation. In the Real group, the amplitude was significantly increased compared to that of the Sham group

According to the brain mapping analysis, the P300 amplitude was found to be increased in all subjects. Although the change in the P300 amplitude was small in the male group, a marked change was observed in the female group. The strongest change in the P300 amplitude was observed in the frontal and parietal lobe areas. In particular, a noticeable increase in the P300 amplitude was detected in the female group (Fig. 3).

### Slow alpha wave power spectrum

The slow alpha waves of the background potential were analyzed separately for the male and female groups, and no significant changes in the mean frequency were observed in either group. However, the mean power decreased at all the analyzed channels in the male group: Fz (F = 0.77, *p* = 0.384), Fcz (F = 1.15, 0.293), Cz (F = 1.54, *p* = 0.227), Cpz (F = 2.52, *p* = 0.126), and Pz (F = 1.78, *p* = 0.195). In contrast, in the female group, the mean power increased at all the analyzed channels: Fz (F = 4.19, *p* = 0.051), Fcz (F = 4.41, *p* = 0.46), Cz (F = 2.30, *p* = 0.141), Cpz (F = 356, *p* = 0.070), and Pz (F = 4.52, *p* = 0.043). In particular, a significant increase was observed at the Fcz, and Pz channels (Table 4).

**Table 4:** Mean power and frequency of the slow alpha waves in the female group

		Female (*n* = 29)						Group difference (AS-NS)	*p*-value
		Sham (*n* = 15)			Real (*n* = 14)				
		Before	After	Difference	Before	After	Difference		
Slow alpha mean frequency (Hz)	Fz	9.31 ± 0.23	9.34 ± 0.19	0.03	9.35 ± 0.23	9.35 ± 0.23	0.00	−0.03	0.94
	Fcz	9.32 ± 0.24	9.33 ± 0.22	0.01	9.37 ± 0.21	9.34 ± 0.22	−0.03	−0.04	0.65
	Cz	9.34 ± 0.23	9.35 ± 0.21	0.01	9.37 ± 0.22	9.35 ± 0.22	−0.02	−0.03	0.50
	Cpz	9.34 ± 0.22	9.36 ± 0.19	0.02	9.39 ± 0.22	9.38 ± 0.22	−0.01	−0.03	0.44
	Pz	9.36 ± 0.23	9.39 ± 0.17	0.03	9.42 ± 0.23	9.40 ± 0.23	−0.02	−0.05	0.61
Slow alpha mean power	Fz	61.11 ± 53.13	41.91 ± 29.97	−19.20	45.34 ± 46.20	45.88 ± 45.65	0.54	19.74	0.05
	Fcz	64.42 ± 54.31	45.89 ± 35.54	−18.53	49.82 ± 52.09	50.84 ± 51.38	1.02	19.55	*0.05*
	Cz	64.43 ± 59.39	51.44 ± 58.03	−12.99	51.91 ± 57.46	52.62 ± 56.64	0.71	13.70	0.14
	Cpz	75.47 ± 99.73	62.11 ± 98.03	−13.36	54.37 ± 62.78	55.64 ± 64.53	1.27	14.63	0.07
	Pz	81.83 ± 124.38	63.86 ± 102.47	−17.97	53.70 ± 64.64	56.12 ± 69.15	2.42	20.39	*0.04*

Similar to the results in the male group, in the female group, there was no significant change in the mean frequency of the slow alpha waves. Unlike in the male group, however, the mean power tended to increase at all the analyzed channels, and significant changes were observed at three channels: Fcz (F = 4.41, *p* = 0.046), and Pz (F = 4.52, *p* = 0.043)

Italicized data: A significant increase in the slow alpha mean power

### Double-blind index

According to the results of subject survey administered immediately following the experiment, 11 subjects thought they had received stimulation, 4 thought they had not, and 13 answered that it was hard to tell in the Real group. In the Sham group, 12, 7, and 8 subjects were in each category, respectively. The presence of actual stimulation and the subject’s perception of the presence of stimulation were tested by a chi-square test and a *p*-value of 0.68 was obtained, indicating that this study was successfully conducted as double-blind (Table 5).

**Table 5:** Double-blind index

	Real (n(%))	Sham (n(%))	Unknown (n(%))	Total (n(%))	*p*-value
Real	11 (20.00%)	4 (7.27%)	13 (23.64%)	28 (50.91%)	**0.68**
Sham	12 (21.82%)	7 (12.73%)	8 (14.54%)	27 (49.09%)	
Total	23()	11()	21()	55()	

The chi-square test results indicate that the blinding was well-maintained, with a *p*-value of 0.68

Bold data: No placebo effect (*p* > 0.05)

## Discussion

Based on previous studies, the physiological significance of the amplitude and latency of the P300 can be summarized as follows: The amplitude of the P300 represents contextual updating of attentional allocation and working memory resources and is proportional to the resource allocation for a specific task or stimulus. The latency represents cognitive processing and classification speed and is inversely proportional to cognitive capability and attentional allocation. In this study, changes in the amplitude and latency of the P300 were used to evaluate the effect of electrical stimulation of the foot on the reduced cognitive function that can develop with aging.

BL62 and KI6 are acupuncture points effective in the treatment of mind-related diseases in oriental medicine. They are known to stabilize mental conditions and to clear the mind, and they have recently been found to be effective in cases of insomnia. Studies of the acupuncture stimulation of acupuncture points have shown that continuous stimulation at BL62 reduced the speed of nerve reactions and that the stimulation of several acupuncture points, including KI6, was effective in treating postmenopausal hot flashes. Few studies of BL62 and KI6 stimulation have been conducted with regard to responses to stimulation or treatment effects, but such studies should be considered because this stimulation has been shown to be effective in the treatment and prevention of insomnia, hypersomnia, and brain disorders.

In this study, low-frequency electrical stimulation of BL62 and KI6, which is easily applied to human patients and is inexpensive and safe, with few adverse reactions, was performed to determine its effect on cognitive function by measuring and analyzing background EEG and ERPs. Using low-frequency electrical stimulation, a double-blind test was performed to prevent any placebo effects, and the objectivity of the study results were confirmed by a chi-square test.

Studies of the P300 generally use closely related channels such as Fz, Cz, and Pz for analysis, but in this study, in addition to those 3, 2 more channels, Fcz and Cpz, were selected among the 32 channels to increase the resolution. For statistical analysis, ANCOVA was performed to compare changes before and after stimulation because brain waves vary greatly in amplitude from individual to individual. Additionally, the stimulation group and the Sham group did not contain the same individuals, and the number of subjects in each group differed; thus, the pre-stimulation values were different.

The latency of the P300 tended to increase at 4 of the channels (Fz, Fcz, Cz, and Pz) in all the subjects, but the differences between the stimulation and non-stimulation groups were not significant. However, the amplitude was increased at all 5 the analyzed channels after stimulation, with particularly significant changes at Cpz.

The results of the male and female subjects were compared in the analysis. In the males, the latency and amplitude did not differ between the stimulation group and the non-stimulation group, but in the females, the amplitude was significantly increased at the channels Fz, Fcz, Cz, Cpz, and Pz in the stimulation group compared to the non-stimulation group. Brain mapping confirmed that the amplitude of the P300 was greatly enhanced in the frontal and parietal lobes in the females.

Studies have long shown prolongation of the latency and diminution of the amplitude of the P300 with increasing age, which suggests that cognitive functions decline, with reduced responsiveness and brain function due to a reduction in brain cell capacity and to neurodegeneration, as an individual ages. The increase in P300 latency and amplitude with low-frequency electrical stimulation of BL62 and KI6 suggests the possibility of delaying these age-related phenomena.

Intriligator et al. [19] reported a relationship between background EEG and the P300, in which the latency of the P300 and the slow alpha mean frequency were both increased, whereas the amplitude of the P300 and the slow alpha mean power were both decreased. Hillman et al. [20] confirmed that vigorous cardiovascular exercise not only increased alpha activity but also increased the amplitude and decreased the latency of the P300. In our study, the analysis of the mean frequency and power of the slow alpha waves showed that in the women, the frequency increased and the mean power tended to increase at all 5 channels after stimulation most significantly at Fcz, and Pz. This result may be related to the increased P300 amplitude at all the channels.

Polich and Criado [3] reported that long-term alcohol intake can reduce the amplitude of the P300, and Kalmijn et al. [21] reported that smoking and alcohol consumption can lead to greater reductions in cognitive function in males than in females. In this study, through an additional survey, it was confirmed that among those subjects who received the stimulus, the males were exposed to more alcohol and engaged in more smoking than the women (Table 6); this may have acted as a negative factor for the improvement of the P300 amplitude.

**Table 6:** Subject ratings of each gender group with respect to drinking, smoking during the study period

	Drinking	Smoking
Male	30.77%	15.38%
Female	23.08%	0%

The male subjects drank and smoked more than the female subjects

The results described above confirmed that the two dimensions of the P300 (latency and amplitude) were improved by long-term, low-frequency electrical stimulation, with a particularly significant increase in the amplitude of the P300. Changes in the amplitude of the P300 are known to be related to the regions responsible for attention and working memory. Thus, it is possible that low-frequency electrical stimulation of Bl62 and KI6 has a positive effect on memory function in middle-aged women and thus may prevent brain aging due to a reduction in brain cell capacity and to neurodegeneration, thereby reducing risk for dementia. However, because this study was performed on subjects in their 50s, additional studies are needed to determine the effects on different age groups and also to assess the stimulation effect in men by minimizing distracting factors. In addition, the pathways by which the stimulation of acupuncture points affects the brain neurologically and the mechanisms that change the two dimensions of the P300 should be further explored through animal experiments.

## Conclusion

This study confirmed that the amplitude of the P300 is increased in middle-aged women by low-frequency electrical stimulation at BL62 and KI6. Thus, the results of this study may serve as a foundation for the treatment of dementia and various brain disorders due to brain aging and impaired cognition. However, because the results of this study are limited to middle-aged women, further studies are necessary to determine the therapeutic effects in different age groups as well as in men and to discover the neurological mechanisms underlying the positive influence of low-frequency electrical stimulation on the brain.

## Abbreviations

CRIS: Clinical Research Information Service
EEG: electroencephalogram
ERP: event-related potential
IRB: Institutional Review Board
